# Does a Better Perfusion of Deconditioned Muscle Tissue Release Chronic Low Back Pain?

**DOI:** 10.3389/fmed.2018.00077

**Published:** 2018-03-20

**Authors:** Paola Valdivieso, Martino V. Franchi, Christian Gerber, Martin Flück

**Affiliations:** ^1^Laboratory for Muscle Plasticity, Department of Orthopedics, University of Zurich, Zürich, Switzerland; ^2^Interdisciplinary Spinal Research, Department of Chiropractic Medicine, Balgrist University Hospital, Zürich, Switzerland; ^3^Orthopedics Department, Balgrist University Hospital, University of Zurich, Zurich, Switzerland

**Keywords:** non-specific chronic low back pain, muscle perfusion, gene polymorphisms, pain sensation, therapeutics

## Abstract

Non-specific chronic low back pain (nsCLBP) is a multifactorial condition of unknown etiology and pathogenesis. Physical and genetic factors may influence the predisposition of individuals to CLBP, which in many instances share a musculoskeletal origin. A reduced pain level in low back pain patients that participate in exercise therapy highlights that disuse-related muscle deconditioning may predispose individuals to nsCLBP. In this context, musculoskeletal pain may be the consequence of capillary rarefaction in inactive muscle as this would lower local tissue drainage and washing out of toxic waste. Muscle activity is translated into an angio-adaptative process, which implicates angiogenic-gene expression and individual response differences due to heritable modifications of such genes (gene polymorphisms). The pathophysiologic mechanism underlying nsCLBP is still largely unaddressed. We hypothesize that capillary rarefaction due to a deconditioning of dorsal muscle groups exacerbates nsCLBP by increasing noxious sensation, reducing muscle strength and fatigue resistance by initiating a downward spiral of local deconditioning of back muscles which diminishes their load-bearing capacity. We address the idea that specific factors such as angiotensin-converting enzyme and Tenascin-C might play an important role in altering susceptibility to nsCLBP *via* their effects on microvascular perfusion and vascular remodeling of skeletal muscle, inflammation, and pain sensation. The genetic profile may help to explain the individual predisposition to nsCLBP, thus identifying subgroups of patients, which could benefit from *ad hoc* treatment types. Future therapeutic approaches aimed at relieving the pain associated with nsCLBP should be based on the verification of mechanistic processes of activity-induced angio-adaptation and muscle-perfusion.

## Overview of the Clinical Manifestation

Low back pain (LBP) is the most common cause for years lived with a disability (1) with 60–80% of the adult population reporting LBP for some time along their lives (2). Problematic is the fact that only 5–15% of all LBP cases can be associated with a specific cause. The majority of patients with LBP present non-specific form, in which no common anatomical origin of the pathology can be identified and matched to the patient’s complaints (3). Non-specific LBP accounts for 70–90% of all LBP cases (3–5). Most LBP cases recover within days or weeks without further complications after first signs of pain manifest (6). However, a minority of about 10% does not recover and develop chronic LBP (CLBP), identified as a condition lasting longer than three months (3, 7). Although CLBP is recognized to present a multifactorial origin, the occurring pain in this group of non-specific chronic low back pain (nsCLBP) is often associated with musculoskeletal disorders (8).

## Muscle Disuse and Deconditioning in nsCLBP

The muscular system is of pivotal importance for the stability and movement of the spine (9). Inappropriate use of local musculoskeletal system blunts the impact of physiological cues which are required to maintain the functional capacity of muscle through instructing homeostatic processes and adaptation (10). As a consequence, the musculoskeletal system is deconditioned and the capacity of stabilize posture and move is compromised (11, 12). On one hand, muscle deconditioning, due to a congenital or acquired lowering of muscle activity can be seen as a cause of nsCLBP. This may cause constant overloading of the lumbar muscles and subsequent increase in muscular fatigue (13, 14) which ultimately may lead to nsCLBP. On the other hand, muscle deconditioning can be regarded as a consequence of CLBP due to a vicious cycle consisting of an initial injury, which may lead to pain and subsequent avoidance behavior regarding physical activities due to the fear of re-injury (15). Such vicious cycle may lead to muscle deconditioning, leaving the patient unable to sustain “normal” everyday loading (16–18). Whether muscle deconditioning represents a possible cause or, instead, consequence of nsCLBP, is still a point of debate (17, 19).

In a recent review, Steele et al. (15) have pointed out that “deconditioning” (i.e., defined by the authors as a “decrease in function”) may not be only caused by “disuse” [defined by Verbunt et al. as a decrease in physical activity levels (17)]. In LBP, trunk muscles concomitantly undergo a specific acute-to-chronic phase remodeling which usually leads to a reduced cross-sectional area (CSA) of involved muscles (20). This phenomenon is well understood to reflect a specific local response of muscle tissue to the reduced impact of use-related metabolic and mechanical cues, or muscle disuse (21).

Muscular disuse is defined as the response to decreased usage, leading to a reductive remodeling of the tissue (22), accompanied with a shift from slow to fast myosin isoforms muscle phenotype (23), markedly decreased force output and fatigue resistance, lower neural activation, reduced metabolic supply due to capillary rarefaction, and a diminished local oxidative capacity (24). This physiological definition of disuse distinguished from the one describing disuse as a “general lack or reduction of physical activity level.” Lower physical activity levels can indeed be associated with local muscle disuse and deconditioning (25), but some authors have found inconclusive relationships between physical activity and CLBP (17), as whole body activity levels seem not to differ between healthy controls and CLBP patients (26). However, in the formed instances physical activity was assessed by accelerometer and average daily metabolic rate to resting metabolic rate ratio (26). As no information on localized (i.e., trunk) muscle activation or energy expenditure was provided, the presence of concomitant local muscle disuse cannot be excluded. Thus, muscle disuse may still be regarded as a cause of nsCLBP pathology. To date, as it is unsolved whether muscle deconditioning is cause or consequence of disuse; there is an urge for longitudinal investigations with clearly defined and standardized measures for conditioning levels (strength and endurance) and strict control of influencing factors (17).

## Indicators of Muscle Deconditioning in nsCLBP

Morphological indicators of muscle deconditioning are provided by imaging and histochemical studies of lumbar musculature. During deconditioning, skeletal muscles go through remodeling processes that lead subsequently to a significant loss in mass (15). After prolonged physical inactivity, paraspinal muscles undergo atrophic processes (27, 28): morphometric estimates based on magnetic resonance imaging (MRI) presented a decrease of CSA in paraspinal muscle, especially the multifidus (20, 29). Similar patterns of atrophy have been demonstrated using ultrasound imaging (30, 31) and computed tomography (CT) (32–34). Although the significance of paraspinal muscle atrophy role in predicting long term of LBP has been challenged (35, 36), there is evidence for a time-dependent selective ipsilateral atrophy of distinct muscle groups specific for the symptomatic side (37, 38). This remodeling process varies between the acute and CLBP phase, which affects psoas major/erector spinae and multifidus/erector spinae, respectively (37). Goubert et al. stated in a recent review that, in nsCLBP patients, multifidus and paraspinal muscle, but not erector spinae, present the most significant reduction in CSA (39). Nonetheless, transverse relaxation time (T2) MRI analyses revealed abnormalities and asymmetries of low back muscles in patients with acute LBP (40) and such alterations in MRI-derived T2 can be associated with muscle (i.e., multifidus) disuse/altered use of multifidus muscle even after cessation of LBP (41).

Magnetic resonance imaging data have been used in order to measure an alteration of imaging signal intensity, which can be regarded as an index of muscle quality and increase in fat content, which in turn has been found associated with LBP (42, 43).

In addition, histological studies have pointed out changes in the fiber type distribution of back muscles, presenting a shift (in paraspinal muscle) toward the fast-fatiguable type IIX fibers (14, 44, 45).

Functional indicators of muscle deconditioning in patients with nsCLBP are represented by weakness of trunk muscles, measured either by isometric or isokinetic contractions (46) and reduced endurance of lumbar extensor muscles (47). There is evidence that individuals with CLBP have a delayed activation of back muscle activity when performing prone hip extension (48). Lower activation patterns (EMG) of multifidus and other lumbar muscles have been showed during sitting in non-specific CLBP patients compared with healthy controls (49).

## Exercise-Training Regimes in nsCLBP: The Story so Far

It is well established that muscle strength and endurance are compromised in consequence of muscle atrophy and deconditioning (50–53). Therapeutic interventions should be identified and applied to mitigate the atrophic process. Previous training interventions were performed in patients with CLBP, in which lumbar multifidus muscle CSA was found to increase after 10 weeks of stabilization training combined with dynamic-static resistance training (32).

However, there are controversial opinions concerning the relationship between improving trunk muscle performance (i.e., strength/endurance) and relief of painful symptoms and disability (54). We identify that this criticism is related to the lack of a specific exercise treatment to halt LBP and evidence for casual relationship between changes in strength or CSA and pain and disability level, respectively (55). The note of a relationship between pain and muscle’s force producing capacity is provided by the fact that a failure of paraspinal muscles in protecting passive spinal and pelvic structures from excessive loading in daily living maneuvers or sport activities may result in damage to these pain-sensitive structures thus elevating noxious influences. For this reason, specific forms of resistance exercise are often recommended for back pain patients. The conditioning of musculature by progressive resistance exercise improves muscle hypertrophy, strength/endurance performance, and cardiovascular capacity (56). In addition, therapeutic training that improves both of the former parameters was found to relieve symptoms of CLBP and disability (54, 57) related to musculoskeletal disorders. There is also evidence for a combination of electrical stimulation or ultrasound therapy and exercises in increasing quality of life by improving pain, muscle strength/endurance and spine mobility (58).

## Muscle Perfusion and nsCLBP

In the late 1700s, a Scottish surgeon (John Hunter) observed that “blood goes to where it is needed” (59). Along this original line of observations it is now understood that the primary effect of mechanical-stimuli such as repeated muscle contractions will produce a significant increase in cardiac output followed by an increase in blood volume. Thus, the vast majority of the increased metabolic and mechanical demand is allocated to the contracting skeletal muscle and adequate muscle perfusion is crucial (60).

Muscle tissue is dependent on sufficient blood perfusion in terms of maintaining oxygen- and nutrient levels according to its demands (61). Insufficiency of both, oxygen- and nutrient levels, as well as insufficient removal of toxic products of the muscle metabolism, causes pain, and deteriorate physiological functioning (62). Thus, capillary regression or rarefaction, caused by physical deconditioning, chronic vasoconstriction, maladaptive alterations/diseases of the microvascular bed, or endothelial dysfunction in muscle tissue (63–65), may lead to muscle ischemia in consequence of an insufficient blood flow and decreased oxygen extraction within the muscle tissue due to an associated reduction in aerobic capacity of muscle fibers (66).

Previous literature highlighted the evidence for a potential connection between impaired perfusion (i.e., atherosclerosis and stenosis of lumbar arteries) and CLBP (67, 68). Other studies showed that an insufficient lower limb perfusion may induce a similar symptom as neuropathic pain (69, 70). Interestingly, prolonged retraction of the paraspinal muscle as induced during spinal surgery may reduce muscle perfusion and trigger back pain and disability (71). Paraspinal compartment syndrome is associated with reduced tissue perfusion that causes ischemic pain (72) as well as disk degeneration (67).

Taken together, these data point to insufficient blood supply, with evidence for hypoxic or ischemic conditions within the lumbar compartment, which may affect the metabolism of vertebral bone, nerve roots, intervertebral disks, and lumbar muscles. Interestingly, some study suggests that CLBP patients present an impairment to deliver oxygen in erector spinae muscle, which can be partly restored during exercise (73–75).

## Muscular Angio-Adaptation and CLBP

Aberrant governance of back muscle strength and endurance through use dependent vascular perfusion may represent a critical event in the etiology and progression of CLBP. In this context, a critical lack of dynamic back muscle work is a possible causal factor leading to muscle deconditioning through rarefaction of capillaries and reduced aerobic capacity (76). Regression of muscle capillaries is a common consequence in physical deconditioning (i.e., hind limb unloading) and chronic diseases such as diabetes, obesity, chronic heart failure, or chronic obstructive pulmonary disease (76, 77). The consequent microcirculatory dysfunction exacerbates the compounds underlying the pathology through further inflammation and impaired delivery of oxygen and substrate to tissues (78, 79). Although the pathophysiologic mechanisms remain unclear, it is known that capillary supply supports the homeostatic processes in muscle tissue: a qualitative and quantitative improvement in muscle perfusion, following arteriolar vasodilatation and growth of the capillary tree, enhances muscle metabolic capacity to augment resistance to fatigue (80, 81). Moreover, a better perfusion could explain analgesia produced by washing out of toxic wastes and reducing muscle and joint stiffness (82).

In normal adult skeletal muscle, the genesis and turnover of capillaries and muscle fibers are inter-related (80, 83, 84). Being skeletal muscle a plastic tissue, under repeated mechanical stimuli, angiogenesis is requested to compensate perfusion distance between capillaries and muscle fibers. Several potential stimuli have been postulated to underlie angiogenesis in exercised muscle. An increase in blood flow within a vessel causes increases in shear stress and lowered oxygenation, which is a major stimulus in the remodeling and capillary growth (85). In fact, abluminal mechanical stress (i.e., stretch) of muscle tissue with static or repeated cycles of muscle contraction represents the physiological growth stimulus for the embedded capillaries which is equally effective as liminal sheer stress to induce growth of the capillary endothelium through the sprouting of existing blood vessels (84, 86). In this process, vasoactive factors initiate and control the remodeling of the basement membrane that surrounds the capillary endothelium by regulating the proliferative activity and motility of endothelial cells (ECs). Capillary remodeling encompasses changes in the connections between the ECs and the extracellular matrix (ECM) proteins through the activation and enhanced synthesis of adhesive and cytoskeletal proteins and extracellular proteases. Molecular control of the angiogenic process is then orchestrated by the interplay between the activation of ECs and turnover of the ECM. There is growing evidence suggesting that matrix-derived factors play a critical role in regulating muscle angiogenesis (87). The endothelium and myogenic control collaborate in vascular adaptation in response to several stimuli during vasodilatation/vasoconstriction responses (88).

## The “Muscle Perfusion” and “Pain” Hypothesis

On the basis of the aforementioned evidence, we develop the idea that individuals with nsCLBP might present an attenuation of muscle-perfusion, due to muscle deconditioning and concomitant disuse. In this scenario, an altered perfusion and rarefaction of capillaries may be the result of a complex interplay between genetic predisposition and environmental cues (such as muscle deconditioning).

Several studies have investigated a possible linkage between the musculoskeletal system and genetic polymorphisms affecting pro- and anti-angiogenic factors, in particular those being associated with the remodeling of the ECM and the proliferation of ECs (89). Perfusion-related angio-adaptation may implicate the regulation of muscle’s vascular bed by two potentially angiogenic modulators, angiotensin-converting enzyme (ACE) and Tenascin-C (TNC).

In a recent investigation, we have illustrated how ACE and (TNC) cooperate during muscular and vascular-remodeling (90). In cultured smooth muscle cells, TNC expression is induced by the vasoconstrictor peptide angiotensin II and is found increased in vascular structures in patients with hypertension (91–93). TNC is highly expressed in migrating ECs and branching sites of blood vessels (91, 94, 95). Suspicion is high that vasoconstriction and remodeling of resistance vessels are somehow connected and that, while TNC mediates the effect of angiotensin II, ACE in turn catalyzes the conversion of angiotensin I to angiotensin II.

**Figure 1:**
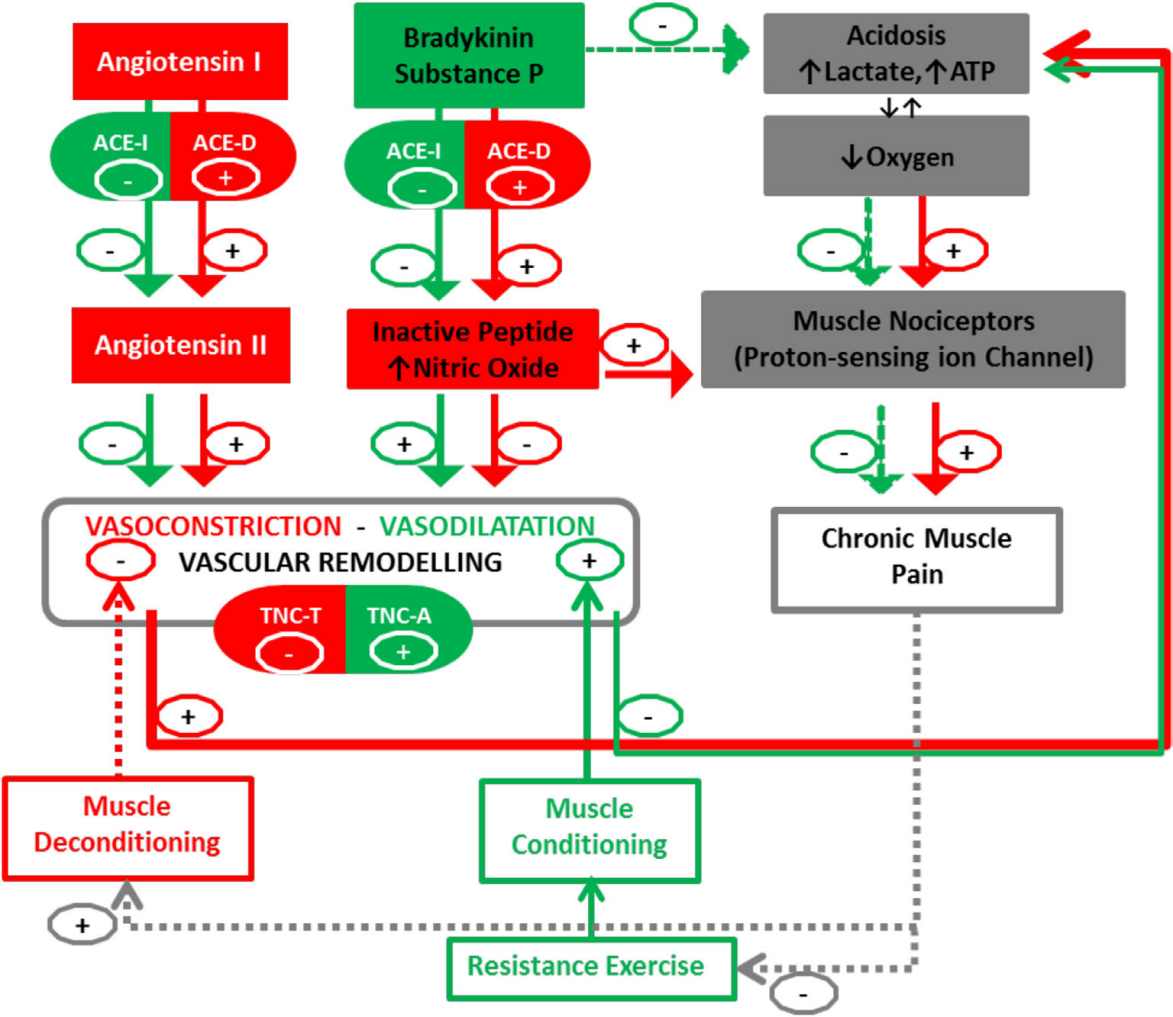
Peripheral blood perfusion in relationship with angiogenic factors (ACE and TNC) and chronic muscle pain. Interactions between muscle deconditioning/conditioning responses and the Angio-adaptation process. Arrows (+) indicate promotion and Arrows (−) indicate reduction. Documented interactions are fully colored in green and red, while dotted lines represent our postulated interactions in this pathway. ACE I, angiotensin-converting enzyme insertion; ACE D, angiotensin-converting enzyme deletion; TNC, Tenascin-C (rs2104772); A, adenine; T, thymine. The TNC-T and ACE-D polymorphism may result in a less-efficient vascular remodeling-perfusion and exacerbation of muscle pain by an increase of nitric oxide (NO) and acidosis-related process.

We then propose that the aforementioned gene polymorphisms, being associated with muscular hypoperfusion due to an enhanced vasoconstriction, may importantly modify the angio-adaptation process. Consequently, we advocate investigating the association between single-nucleotide polymorphisms (SNPs) within the ACE and TNC genes together with changes in morphologic and functional characteristics of skeletal muscle, and clinical outcomes (pain, disability) in patients with nsCLBP in response to therapeutic exercise training (Figure 1, for a synopsis). The proposed mechanism may then operate in two distinct ways: on one hand, vasoconstriction will be regulated by ACE-I/D polymorphism, with the presence (I-allele) or the absence (D-allele) in intron 16 of the ACE gene. Presence of the I-allele may then lower ACE activity in skeletal muscle and the production of Angiotensin II (96, 97). On the other hand, remodeling will also be associated with polymorphism in the TNC gene (98). In particular, rs2104772 polymorphism describes a thymidine (T)-to-adenosine (A) exchange at nucleotide position 44513 in the TNC gene that instructs the substitution of leucine by isoleucine at amino acid position 1677 and it may alter the molecular elasticity of the TNC Fn-III-D domain (99, 100). Speculatively, *via* this molecular effect, the TNC T-allele cooperates with the ACE D/D modulated vasoconstriction to blunt vascular remodeling and increased perfusion in back muscles after exercise training compared with other polymorphisms. There is evidence for a direct modulatory connection between the ACE enzyme and pain sensation through modification of the balance between the vasoconstrictive angiotensin peptide and pain-modulatory and vasodilatatory peptides of the kinin family, bradykinin and substance P (101, 102). For instance, ACE degrades substance P into its inactive form and ACE-regulated vasoconstriction exacerbates muscle pain by an acidosis-related process (103). There is in fact mounting evidence that acidosis could activate muscle pain sensation mediated by activation of acid-sensing ion channels (104) and there is mounting evidence that proton-sensing receptors are involved in the Transition from Acute to Chronic Pain (105).

Intriguingly, substance P signaling was recently pointed out to exert an anti-nociceptive role through modulation of activation potential threshold in certain muscle nociceptors as well (103). While the underlying patho-physiological process remains to be disentangled, ACE-mediated inhibition of substance P emerges as a potentially important factor affecting pain sensation in nsCLBP.

However, there is evidence that repeated nociceptive stimuli can alter peripheral and/or central sensitization, resulting in the chronicity of pain with both nociceptive and neuropathic pain components (106). Combination pharmacotherapy is often necessary in patients with CLBP, in order to manage both nociceptive and neuropathic pain (107).

It is noteworthy to highlight that the first line therapy in CLBP recommend the use of vasoactive drugs. Acetaminophen (paracetamol) and non-steroidal anti-inflammatory drugs “NSAIDs” or opioids are common drugs and have widespread use for acute and chronic musculoskeletal disorders. The advantage is a peripheral effect in alleviating ischemic pain by independent mechanisms of vasodilation that increase blood flow in nerve and in muscle tissue.

## Clinical Relevance and Future Direction

The postulated connection of CLBP and lower muscle perfusion provides a conceptual framework to develop a treatment that tackles the pain generating mechanism with a personalized approach.

Toward this end, studies should first investigate the association between CLBP and polymorphisms for use-dependent angiogenic factor expression that may predict the CLBP pathology. Successively, investigations should gain insights into such polymorphisms and their significant role in vascular muscle remodeling in response to exercise training. From here, potential tailored exercise programs should be identified, aimed at improving strength and fatigue resistance of the back muscles in order to likely protect or minimize the effects of injurious events and reduce sensitivity to pain.

## Author Contributions

PV conceived the manuscript. PV, MFr, and MFl contributed to the writing of the manuscript. PV, MFr, CG, and MFl edited and produced the final version of the manuscript.

## Conflict of Interest Statement

The authors declare that the research was conducted in the absence of any commercial or financial relationships that could be construed as a potential conflict of interest. The reviewer LT and the handling editor declared their shared affiliation.
